# Multi-Modal Proteomic Analysis of Retinal Protein Expression Alterations in a Rat Model of Diabetic Retinopathy

**DOI:** 10.1371/journal.pone.0016271

**Published:** 2011-01-13

**Authors:** Heather D. VanGuilder, Georgina V. Bixler, Lydia Kutzler, Robert M. Brucklacher, Sarah K. Bronson, Scot R. Kimball, Willard M. Freeman

**Affiliations:** 1 Department of Pharmacology, Penn State College of Medicine, Hershey, Pennsylvania, United States of America; 2 Department of Cellular & Molecular Physiology, Penn State College of Medicine, Hershey, Pennsylvania, United States of America; University of Oldenburg, Germany

## Abstract

**Background:**

As a leading cause of adult blindness, diabetic retinopathy is a prevalent and profound complication of diabetes. We have previously reported duration-dependent changes in retinal vascular permeability, apoptosis, and mRNA expression with diabetes in a rat model system. The aim of this study was to identify retinal proteomic alterations associated with functional dysregulation of the diabetic retina to better understand diabetic retinopathy pathogenesis and that could be used as surrogate endpoints in preclinical drug testing studies.

**Methodology/Principal Findings:**

A multi-modal proteomic approach of antibody (Luminex)-, electrophoresis (DIGE)-, and LC-MS (iTRAQ)-based quantitation methods was used to maximize coverage of the retinal proteome. Transcriptomic profiling through microarray analysis was included to identify additional targets and assess potential regulation of protein expression changes at the mRNA level. The proteomic approaches proved complementary, with limited overlap in proteomic coverage. Alterations in pro-inflammatory, signaling and crystallin family proteins were confirmed by orthogonal methods in multiple independent animal cohorts. In an independent experiment, insulin replacement therapy normalized the expression of some proteins (Dbi, Anxa5) while other proteins (Cp, Cryba3, Lgals3, Stat3) were only partially normalized and Fgf2 and Crybb2 expression remained elevated.

**Conclusions/Significance:**

These results expand the understanding of the changes in retinal protein expression occurring with diabetes and their responsiveness to normalization of blood glucose through insulin therapy. These proteins, especially those not normalized by insulin therapy, may also be useful in preclinical drug development studies.

## Introduction

Approximately 97% of patients with Type 1 diabetes develop some degree of diabetic retinopathy (DR) within 25 years of diagnosis [1]. Even with improvements in therapies for glycemic control, DR remains a leading cause of new cases of adult blindness worldwide [2]–[4]. With both manifest and subclinical DR, visual impairment in hue discrimination and contrast sensitivity, delayed dark adaptation, abnormal visual fields and decreased overall visual acuity are observed [5]–[9]. Clinically, the sequence of events in the pathogenesis of DR and the nature of interplay of vascular dysfunction, dysregulated metabolism, and neuronal damage remain to be determined [10].

Research using rat models of diabetes have contributed to the concept of diabetic retinopathy as a progressive neurovascular complication that includes vascular, inflammatory, and neuronal components [11]–[14]. In previous reports, we have identified retinal transcriptomic changes with diabetes associated with vascular dysfunction, a pro-inflammatory state, and neuronal compromise [12], [14], [15].

Reports detailing the retinal proteome in human subjects with DR or animals models of DR are limited. The difficulty in obtaining human retina samples has led to studies using vitreal protein collected from subjects. These studies have provided novel and important insights including the potential role of carbonic anhydrase in vascular leakage [16], [17]. Other human studies of vitreous have identified additional targets for investigation including Complement proteins [18], acute phase proteins [19], Apolipoprotein A1 [20], and interphotoreceptor retinoid-binding protein [21]. These studies provide valuable new insights but do not directly address proteomic changes occurring in the retina. Availability of human post-mortem retinal samples is limited, but animal model systems offer the potential to examine diabetes-induced alterations in retinal protein expression. There are a relatively limited number of reports examining the retinal proteome with diabetes. Studies using rat [22]–[24] and mouse models [25] have identified a number of novel changes in the diabetic retina but have generally relied on a single proteomic approach and have not confirmed discovery findings with independent techniques in multiple animal experiments.

**Figure 1:**
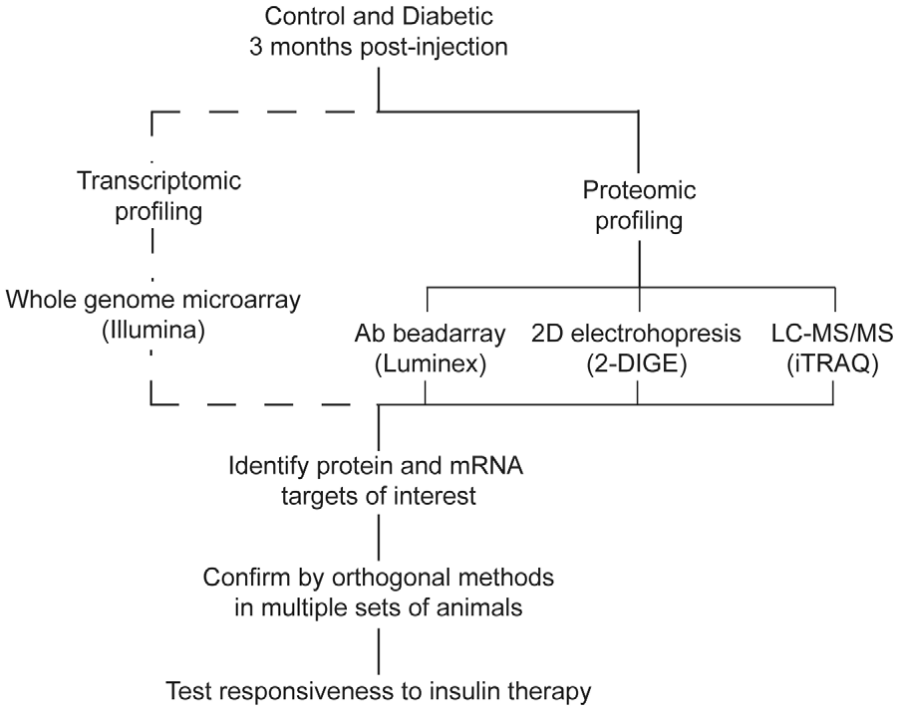
Study design. To provide the widest proteomic coverage possible, three different proteomic discovery approaches (iTRAQ, DIGE, and Luminex) were used. In addition to proteomic discovery analyses, whole-genome transcriptomic analysis was performed. Following data integration, mRNA and protein validation studies were performed in independent animal sets. Lastly, confirmed changes were examined for responsiveness to insulin therapy.

The present study used a multi-modal proteomic discovery approach (Figure 1). A number of quantitative proteomic techniques are currently in use, but no single method provides comprehensive proteomic coverage and previous reports have suggested that individual methods preferentially detect proteins with certain biophysical characteristics [26]. Therefore, this study used Luminex [27], DIGE [28], and iTRAQ [29] methods. These three approaches use distinct methods of both protein separation (Luminex – antibody affinity; DIGE - electrophoretic separation of intact proteins; iTRAQ – liquid chromatography of peptides) and quantitation (Luminex – antibody binding; DIGE – imaging of fluorescently-labeled intact proteins, iTRAQ – MS/MS quantitation of tagged peptides). These results were combined with transcriptomic data to identify targets for confirmation in independent animals and after insulin therapy. Changes in retinal protein and mRNA expression were examined in the streptozotocin-induced Sprague Dawley rat after three months of diabetes. Previously we have determined that in this model, early pathological features of DR, including retinal apoptosis and vascular permeability, are evident at this, but not earlier, time points [14]. We therefore selected this timepoint as a duration of diabetes with clinically-relevant pathophysiological characteristics.

## Results

### Animal Data

Five independent experiments were performed in this study. The rat biometric data are summarized in Table 1. At harvest (3 months after STZ or vehicle injection) diabetic rats were hyperglycemic and underweight in comparison to controls as with previous assessments [12], [14]. Glycosylated hemoglobin levels (% HbA1c) were significantly increased in diabetic animals compared to controls as well (Experiments 3 & 4). In Experiment 5, treatment with subcutaneous slow-release insulin pellets for the second 1.5 months of the 3 month diabetic period significantly reduced blood glucose levels compared to untreated diabetics (p<0.001, SNK post-hoc) to levels not significantly different from controls. Diabetic animals receiving insulin weighed more than untreated diabetic animals but were still significantly lower in weight than controls (p<0.001, SNK post-hoc, in both comparisons). Insulin treatment also reduced HbA1c levels significantly compared to untreated diabetic animals but HbA1c levels remained slightly elevated compared to controls (p<0.001, SNK post-hoc, in both comparisons).

**Table 1:** Animal data for each experiment.

Experiment	Condition	n	Weight (g)	Blood Glucose (mg/dL)	HbA1c (%)	Analyses
1	Control	8	529±12	72±4	Not Assayed	Luminex, DIGE, iTRAQ
	Diabetic	8	261±17*	333±17*	Not Assayed	
2	Control	5	610±57	78±9	Not Assayed	Microarray
	Diabetic	5	311±39*	409±39*	Not Assayed	
3	Control	9	591±18	94±4	3.4±0.1	Immunoblot, qPCR
	Diabetic	9	316±17*	371±17*	7.9±0.5*	
4	Control	10	551±71	127±20	3.4±0.4	Immunoblot
	Diabetic	10	280±57*	487±71*	7.6±1.9*	
5	Control	8	575±53#	132±11	3.5±0.1#	Immunoblot, qPCR
	Diabetic	8	311±40*#	496±41*	9.0±0.8* #	
	Diabetic+Insulin	8	407±44*	117±72*	5.3±1.4*	

Data are presented as mean ± standard deviation.

*p<0.001 vs. Control;

#p<0.001 vs. Diabetic+Insulin.

### Luminex

Of the 59 growth factors, cytokines, and signaling molecules examined in the directed Luminex analysis 46 were observed at detectable levels (Table 2). Four proteins had significant differences in abundance between diabetic and control groups (t-test, two-tailed). Fgf2, haptoglobin, and IL-4 levels were elevated in diabetic animals, while IL-17 levels were significantly decreased in diabetic levels. Due to the small magnitude of differences for IL-4 and IL-17, confirmation analysis was focused on Fgf2 and haptoglobin. The full Luminex data set is available in Table S1.

**Table 2:** Luminex Results.

Protein	Diabetic/Control	p value	Protein	Diabetic/Control	p value
**Apo A1**	1.2		**IL-6**	0.9	
**CRP**	1.2		**IL-7**	1.0	
**EGF**	0.9		**IP-10**	1.1	
**Endothelin-1**	1.1		**LIF**	0.9	
**Eotaxin**	1.5		**Lymphotactin**	1.2	
**Factor VII**	1.1		**MCP-3**	1.1	
**FGF-9**	1.1		**M-CSF**	1.1	
**FGF-2**	2.0	***	**MIP-1alpha**	1.4	
**Fibrinogen**	1.0		**MIP-1beta**	1.0	
**GCP-2**	0.9		**MIP-1gamma**	1.0	
**GM-CSF**	1.0		**MIP-2**	0.9	
**Haptoglobin**	1.5	**	**MIP-3beta**	1.0	
**IFN-gamma**	1.3		**Myoglobin**	1.1	
**IgA**	1.1		**OSM**	1.0	
**IL-10**	1.1		**RANTES**	1.1	
**IL-12p70**	1.0		**SAP**	1.1	
**IL-17**	0.8	*	**SCF**	1.0	
**IL-18**	1.0		**SGOT**	1.1	
**IL-1alpha**	1.1		**TIMP-1**	1.0	
**IL-1beta**	1.0		**Tissue Factor**	1.1	
**IL-2**	1.0		**TNF-alpha**	0.9	
**IL-3**	1.1		**TPO**	0.9	
**IL-4**	1.2	*	**VEGF**	1.0	

46 of the 59 proteins in the assay were detected within the range of the standards. Four proteins demonstrated significant differences between control and diabetic groups.

*p<0.05,

**p<0.01,

***p<0.001, two-tailed t-test, n = 8/group.

### DIGE

**Figure 2:**
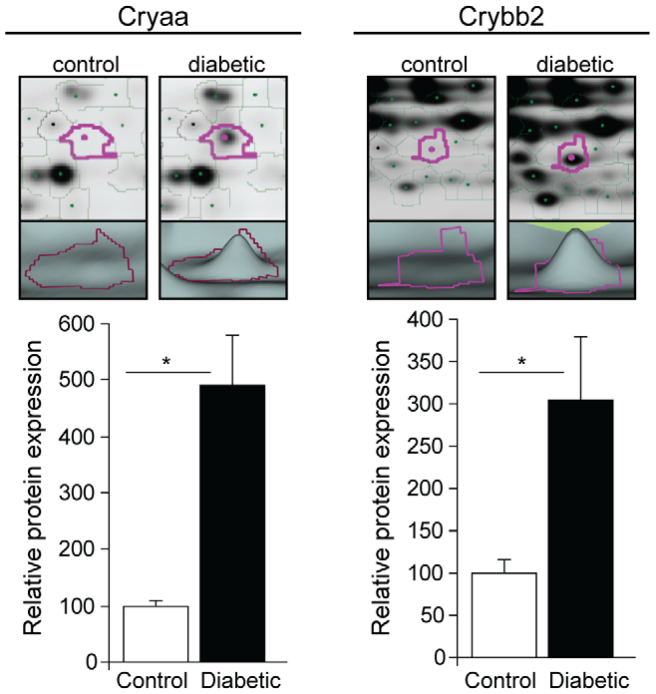
Examples of differentially expressed proteins identified by DIGE. Retinal protein isolated from three-month diabetic and age-matched control rats was separated and quantitated by DIGE analysis with identification by MS/MS. Two examples of confidently identified, differentially regulated protein spots are shown. The upper panels depict spot location and signal intensity, while the lower panels depict three-dimensional representations of the densitometric spot volumes used for quantitation. Both Cryaa and Crybb2 crystallin isoforms were significantly increased in expression (by 491% and 305%, respectively) in diabetic rats compared to controls. * p<0.05, two-tailed t-test, n = 8/group.

Labeled retinal proteins from control and diabetic rats were separated by isoelectric point and molecular weight, producing consistent spot patterns on analytical and preparative gels (Figure 2). 1231 protein spots were matched across >6 analytical gels, yielding n = 6–8/group for differential expression analyses. After subtracting local background and normalizing to Cy-2 signals for inter-gel comparisons, 48 protein spots were determined to be significantly different between control and diabetic rats (≥1.1-fold change, two-tailed t-test, p<0.05). Differential protein expression represented both inductions and reductions with diabetes, ranging from approximately −200% to 500% of control levels.

368 protein spots, including all spots matched to those with significant differential expression and a selection of other spots, were picked for MS-MS/MS protein identification. 288 proteins spots were confidently identified, including 32 of the 48 protein spots with significant differential expression and a fold change ≥1.1 or ≤0.9 (Table 3). Sixteen significantly up-regulated and 16 down-regulated proteins were confidently identified and included several crystallin isoforms, signaling molecules and metabolic enzymes. Aldolase, Cryaa, Crybb2, and Hspa1b were chosen for confirmation analysis. The observation of altered retinal crystallin expression with diabetes confirms our previous findings [23] in this model and we therefore expanded our confirmation analysis to include crystallin isoforms Cryab and Cryba2. The full DIGE data set is available in Table S2.

**Table 3:** Significant protein differences identified by DIGE analysis.

Protein AC#	Protein Name	Symbol	Diabetic/Control	p-value
19526477	Crystallin; alpha A	Cryaa	4.91	*
19526477	Crystallin; alpha A	Cryaa	4.35	*
3891675	Crystallin, beta B2	Crybb2	3.05	*
66730521	Glutathione S-transferase 8	Gsta2	1.47	**
13928850	3-phosphoglycerate dehydrogenase	Phgdg	1.30	**
16758348	Peroxiredoxin 6	Prdx6	1.29	*
1373230	Adenosine kinase	Adk	1.26	**
56090564	Galactose mutarotase	Galm	1.26	*
52345435	Adenosine kinase	Adk	1.19	*
81884653	Aminoacylase-1A	Acy1	1.16	*
47059179	Heat shock 70 kD protein 1B	Hspa1b	1.16	*
347019	DnaK-type molecular chaperone hsp72	Hsp72	1.14	**
40254595	Dihydropyrimidinase-like 2	Dpysl2	1.13	*
51092266	Hypoxanthine guanine phosphoribosyl transferase	Hprt1	1.12	*
13242237	Heat shock protein 8	Hspa8	1.10	*
58865398	Leucine aminopeptidase 3	Lap3	1.10	*
13096481	S- Adenosylhomocysteine Hydrolase	Ahcy	0.90	*
40254595	Dihydropyrimidinase-like 2	Dpysl2	0.89	*
25742763	Heat shock 70 kD protein 5	Hspa5	0.89	*
8393706	Lactate dehydrogenase A	Ldha	0.88	*
16757994	Pyruvate kinase	Pkm2	0.88	*
114326546	Phosphoglycerate mutase 1	Pgam1	0.88	*
8393706	Lactate dehydrogenase A	Ldha	0.86	*
202837	Aldolase A	Aldoa	0.85	**
203078	Nucleolar protein B23.2	Npm1	0.85	*
114326546	Phosphoglycerate mutase 1	Pgam1	0.85	*
6680045	Guanine nucleotide-binding protein; beta-1 subunit	Gnao1	0.84	*
19924089	Nucleoside diphosphate kinase A	Nme1	0.83	***
8393706	Lactate dehydrogenase A	Ldha	0.83	*
56090293	Pyruvate dehydrogenase beta	Pdhb	0.83	*
19526477	Crystallin; alpha A	Cryaa	0.78	*
204444	Guanine nucleotide-binding protein alpha subunit	Gnb1	0.74	*

Thirty-two protein spots with significant differences (t-test, *p<0.05, **p<0.01, ***p<0.001) between diabetic and control groups were identified with high confidence in the mass spectrometry analysis.

### iTRAQ

**Figure 3:**
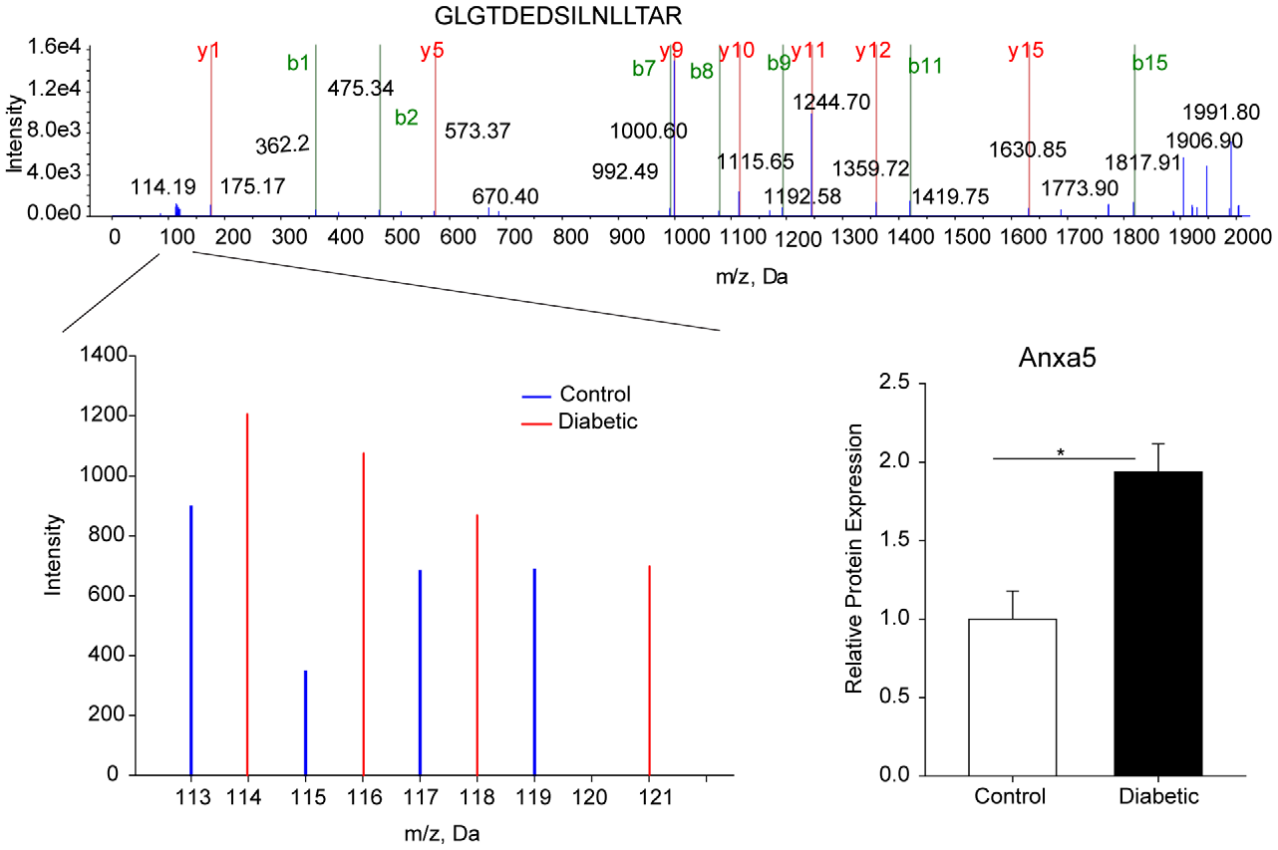
Examples of a differentially-expressed protein identified by iTRAQ. Retinal protein isolated from four diabetic and four age-matched control rats was labeled with isobaric tags prior to separation, identification, and quantitation by 8-plex iTRAQ using LC/MS/MS. An example MS/MS spectrum and the corresponding quantitation data (magnification of iTRAQ signal region) for a confidently identified (>95%) peptide component of Anxa5 are depicted. The signal intensities of all confidently identified peptides unique to Anxa5 were compiled to quantify Anxa5 expression. Anxa5 was significantly increased by approximately two-fold in three-month diabetic rats compared to controls. * p<0.05, two-tailed t-test, n = 4/group.

In the quantitative LC-MS/MS analysis with isobaric tags, 455 proteins were confidently detected with at least two peptides identified with greater than the 95% confidence required for accurate quantitation. A total of 27 proteins were significantly regulated (t-test, two tailed) with a fold change filter of ≥1.1 or ≤0.9. Differential expression was distributed equally between down- (14 proteins) and up- (13 proteins) regulated species (Table 4). Proteins exhibiting expression differences included signaling, cytoskeletal, and neuronal proteins. An example of iTRAQ data is provided in Figure 3 with a representative MS/MS spectrum and quantitation data for a confidently identified peptide component of Anxa5. Anxa5, Hspa1b, Marcks, Dbi, Gnat1, and Rcn were chosen for confirmational analysis. The finding of reduced VAMP2 protein expression after three months of hyperglycemia agrees with our previous observations in this model [13]. The full iTRAQ data set is available in Table S3.

**Table 4:** Significant protein differences identified by iTRAQ analysis.

Protein AC#	Protein Name	Symbol	Diabetic/Control	p-value
55977739	Heat shock 70 kDa protein 1/2	Hspa1b	2.02	*
157830218	Annexin V	Anxa5	1.94	*
78103212	Thymosin beta-4	Tmsb4x	1.56	*
576438	Glutathione transferase A3	Gsta3	1.53	**
266495	Myristoylated alanine-rich C-kinase substrate	Marcks	1.51	*
54261671	Diazepam binding inhibitor	Db1	1.41	*
76779404	Tubulin, beta 2b	Tubb2	1.27	*
81884348	Leucine aminopeptidase	Lap3	1.20	*
81910041	WD repeat-containing protein 1	Wdr1	1.13	*
66793366	Glutamyl-prolyl-tRNA synthetase	Eprs	1.13	*
47115521	COP9 constitutive photomorphogenic homolog subunit 2	Caps2	1.13	*
300798020	CDGSH iron sulfer domain-containing protein 2	Cisd2	1.10	*
109500010	Similar to H2A histone family, member V isoform 1	LOC685909	1.10	*
61098195	RAB3A	Rab3a	0.88	**
94730428	Synaptotagmin-1	Syt1	0.88	**
82546819	Heterogeneous nuclear ribonucleoprotein F	Hnrnpf	0.88	*
55727	Snap91 synaptosomal-associated protein 91	Snap91	0.88	**
157822587	Phosphodiesterase 6B	Pde6b	0.86	*
6978966	Erythrocyte protein band 4.1-like 3	Epb14.3	0.86	*
547890	Microtubule-associated protein 2	Map2	0.80	*
195539900	Tubulin, beta 4	Tubb4	0.73	*
6981614	Vesicle-associated membrane protein 2	Vamp2	0.71	*
83305012	Neurofilament heavy polypeptiden	Nfh	0.68	*
58865424	ATPase, H+ transporting, lysosomal 38 kDa, V0 subunit d1	Atp6v0d1	0.63	*
56054	Dynamin 1	Dnm	0.60	*
158635952	Rod-type transducin alpha subunit	Gnat1	0.52	*
8394171	Reticulocalbin 2	Rcn	0.06	*

Twenty-seven proteins with significant differences (t-test, *p<0.05, **p<0.01) between diabetic and control groups were identified in the LC-MS/MS analysis.

### Transcriptomic

To complement the proteomic analyses, transcriptomic analysis was performed. In the whole-genome transcript analysis of samples from Experiment 2, 11,874 of 22,517 probes on the microarrays met criteria for detection in at least one of the two groups. Within groups, highly consistent gene expression was observed. Examining pair-wise correlations between individual animals across all detected probes, the r^2^ was >0.989 for all control comparisons and >0.982 for all diabetic comparisons. A total of 1274 changes (p<0.05, >1.2 fold magnitude change) were observed, with 583 up-regulated and 691 down-regulated probes as illustrated by a heatmap schematic (horizontal brackets) (Figure S1). The full listing of differentially expressed mRNAs is available in Table S4. As expected, informatic analysis of gene expression pattern similarity clustered all control animals together and all diabetic animals together (vertical brackets). Transcriptomic data were validated by detection of significant changes in 12 of the 14 previously described transcriptomic markers of retinopathy [12], with the only two exceptions showing consistent ratios to previous studies but failing to reach statistical significance. Additionally, a number of acute phase targets identified previously [30] were also identified in this analysis. To extend these transcriptomic findings, Stat3, Cp, and Lgals3 were chosen for confirmation at the level of protein expression. The full complement of microarray data is available through the Gene Expression Omnibus under accession# GSE24423.

### Informatics

**Figure 4:**
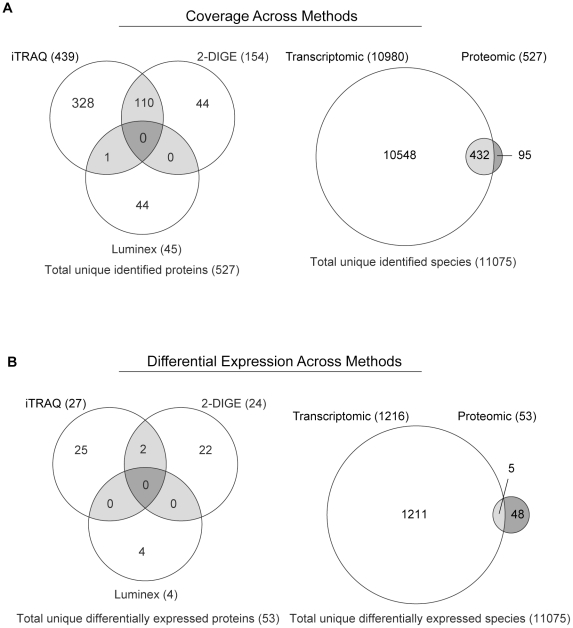
Coverage and differential expression across analysis methods. Bioinformatic comparison of datasets generated by the three proteomic and one transcriptomic approaches used was performed to compare commonalities and differences in coverage and differential expression. (**A**) Comparison of proteomic coverage of unique species identified across methods is illustrated by Venn diagram. iTRAQ, DIGE and Luminex provided complementary coverage with limited overlap between methods. Of the 527 unique proteins identified in this study, 110 were identified by both iTRAQ and DIGE, and only one protein included in the directed Luminex analysis was also identified by an open-profiling method. For the majority of identified proteins, corresponding transcripts were present at detectable level in the transcriptomic analysis. The greater coverage of the transcriptomic data compared to the proteomic coverage is primarily due to the greater sensitivity of the whole-genome microarray approach. (**B**) Comparison of differentially expressed species detected by each method is depicted. As with proteomic coverage, quantitative data proved complementary across methods, with a different subset of differentially expressed proteins detected by each of the three proteomic approaches. In the two cases where a protein was differentially-expressed in both iTRAQ and DIGE quantitation, the direction and magnitude of change were comparable. Numerous differentially-expressed transcripts were identified by microarray analysis, although only a portion of the proteomic changes were detected at the transcript level.

Complementarity of the multi-modal proteomic approach used here was assessed by comparing identities and biophysical properties of unique species detected/identified by technology. Overlap in proteomic coverage was limited (Figure 4A), with no commonality between DIGE and Luminex, and only one species (Got1) detected by both iTRAQ and Luminex. Non-directed approaches demonstrated more overlap, with 110 unique protein products identified by both DIGE (154 proteins total) and iTRAQ (439 proteins total). As expected, the majority (>80%) of species detected/identified by proteomic approaches were also detected at the transcriptomic level by high-density microarray analysis (Figure 4A). Commonality between methods at the level of differential expression was much more limited, with little overlap between proteomic methods. Only Hspa1b and Lap3, which were determined to be up-regulated with diabetes by both DIGE and iTRAQ, were observed by multiple methods (Figure 4B). Importantly, this limited overlap was not due to a protein being observed as differentially expressed by one proteomic method and unchanged by another. Rather, the differences in proteins identified as differentially expressed were largely observed by only one method. Comparison of differentially-expressed species identified by proteomic and transcriptomic analyses demonstrated that few (<10%) changes in protein expression were also observed in the mRNA analysis. While this comparative analysis of the different methods is illuminating it should be noted that the same number of samples were not used in each analysis due to differences in the techniques resulting in slightly differing levels of statistical power.

Potential reasons for the differential coverage of the proteome between proteomic methods are inherent differences in the techniques that may select for proteins with certain biophysical properties (though this is less likely with the Luminex approach in which the analytes are selected). The general biophysical properties of proteins identified by each proteomic approach were similarly distributed across molecular weight and GRAVY score ranges (Figure S2). In each method, detection/identification rates were highest in the 10–40 kDa range and for hydrophilic proteins (indicated by negative GRAVY scores), although proteins identified by iTRAQ tended to be more hydrophilic while DIGE provided slightly more coverage of hydrophobic proteins.

### Confirmation of discovery findings in independent experiments

**Figure 5:**
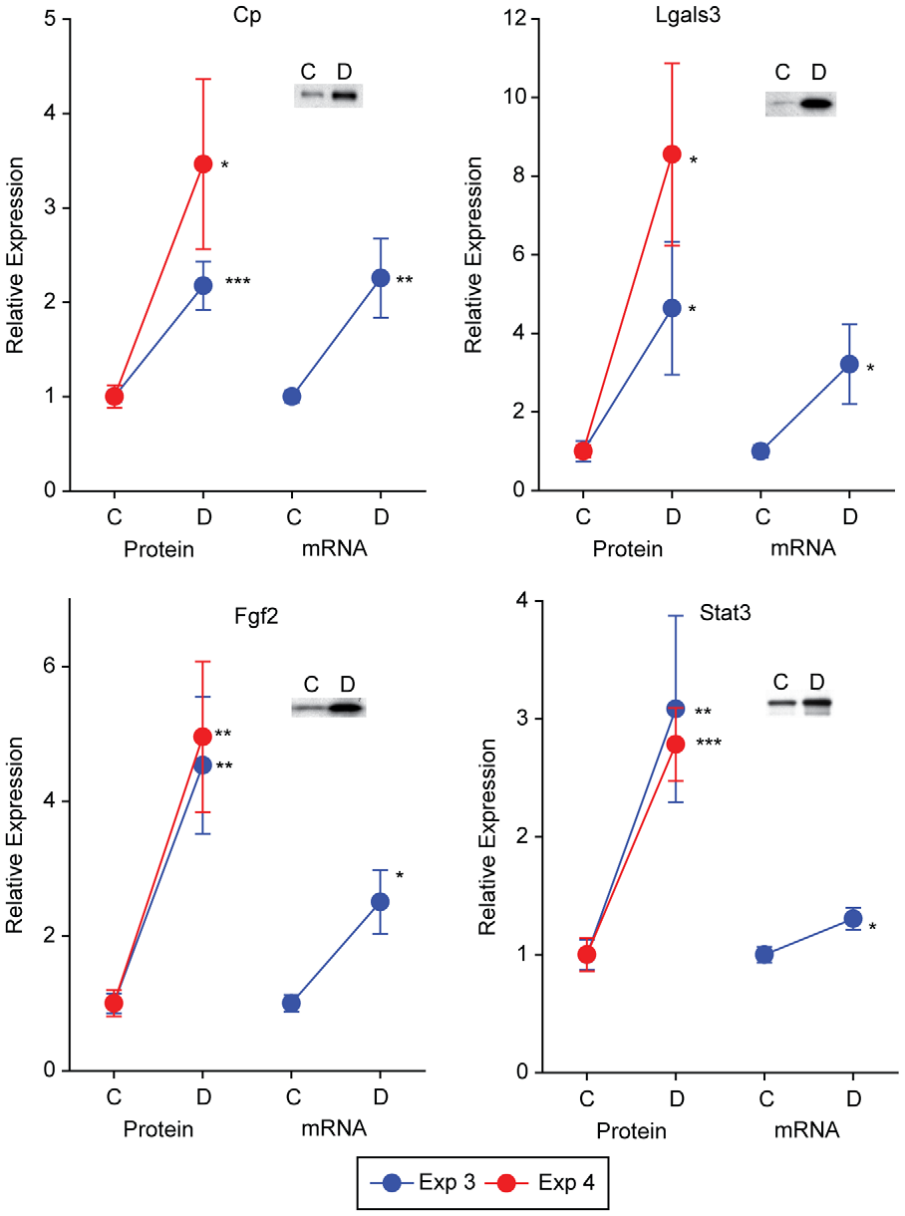
Confirmation of discovery findings in independent animal sets. Targets identified in the proteomic and transcriptomic analyses were confirmed at the level of protein expression by immunoblotting in two independent animal sets (Experiments 3 & 4). mRNA expression was also examined in samples from Experiment 3 by qPCR. Elevated levels of retinal Ceruloplasmin (Cp), Galectin 3 (Lgals3), fibroblast growth factor 2 (Fgf2), and signal transducer and activator of transcription 3 (Stat3) protein were confirmed in both sets of animals. For these proteins the increase in protein expression was mirrored by increased mRNA expression. C = Control, D = Diabetic, * p<0.05, ** p<0.01, *** p<0.001, two-tailed t-test.

**Figure 6:**
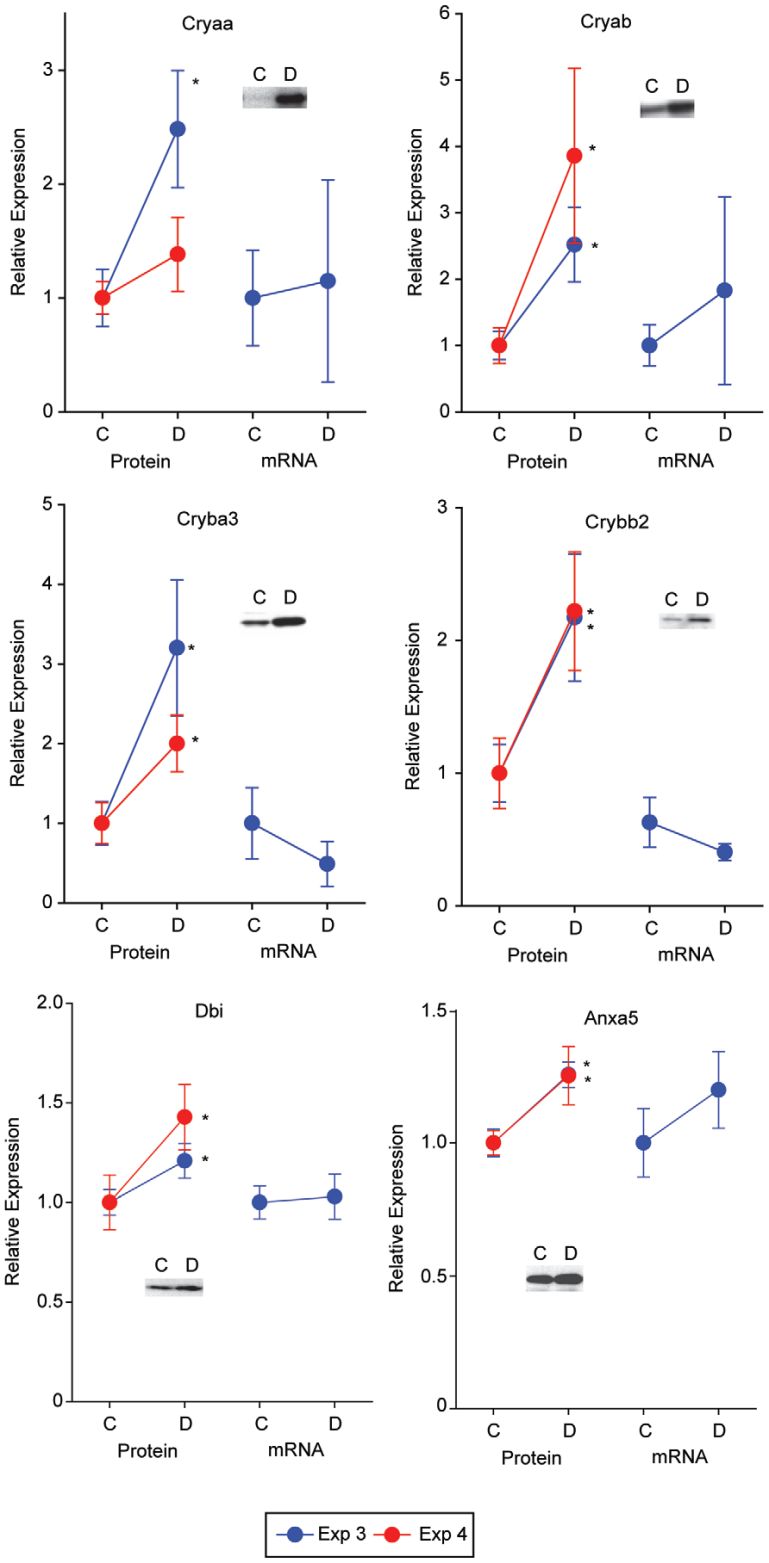
Confirmation of discovery findings in independent animal sets. Targets identified in the proteomic and transcriptomic analyses were confirmed at the level of protein expression by immunoblotting in two independent animal sets (Experiments 3 & 4). mRNA expression was also examined in samples from Experiment 3 by qPCR. Elevated levels of retinal crystallin, alpha B (Cryab); crystallin, beta A3 (Cryba3), crystallin, beta B2 (Crybb2), diazepam-binding inhibitor (Dbi), and annexin 5 (Anxa5) protein were confirmed in two independent animal sets. Crystallin, alpha A (Cryaa) was significantly elevated in one set of animals but did not reach significance in the other. For these six proteins there was no concomitant change in mRNA expression. C = Control, D = Diabetic, * p<0.05, two-tailed t-test.

To confirm discovery findings, immunoblot confirmation analysis was performed using two sets of animals, independent from the discovery experiments (Experiments 3 & 4, Table 1) on selected targets generated from each discovery method. To complement the protein confirmations, mRNA target expression was also examined in the Experiment 3 samples. Targeted protein and mRNA quantitation exhibited high confirmation rates of statistically significant differential expression in both validation experiments. Two patterns of expression were evident with one set of proteins regulated at both the transcript and protein levels (Cp, Lgals3, Fgf2, and Stat3) (Figure 5) and another set with altered protein expression but no significant changes in transcript expression (Cryaa, Cryab, Cryba3, Crybb2, Dbi, and Anxa5) (Figure 6). In all cases the significant differences in protein or mRNA agreed with the discovery findings. Targets from each of the discovery findings (Luminex - Fgf2; DIGE - Cryaa, Crybb2; iTRAQ - Anxa5, Dbi; microarray - Cp, Lgals3, Fgf2, and Stat3) were confirmed by orthogonal methods suggesting the quantitative validity of the discovery methods and reproducibility of the animal model.

Confirmation experiments of haptoglobin, Hspa1b, Marcks, Gnat1, and Rcn did not reveal specific protein detection at the appropriate molecular weight with the antibodies tested. Aldolase was specifically detected but protein expression levels were unchanged (data not shown).

### Effect of insulin treatment

**Figure 7:**
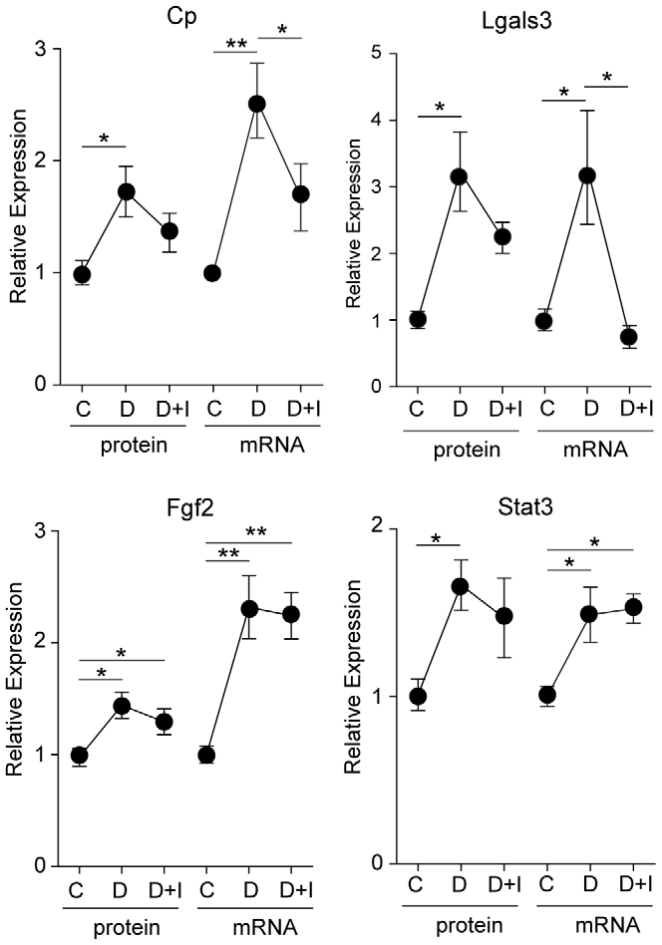
Responsiveness of protein expression to insulin therapy. To examine the effect of insulin treatment on protein and mRNA expression of targets confirmed in the previous experiments, an independent animal experiment was performed with the addition of an insulin-treated diabetic group that received insulin for 1.5 months after 1.5 months of diabetes without insulin therapy. Increased retinal protein and mRNA expression of Cp, Lgals, Fgf2, and Stat3 was observed. Partial normalization of protein expression by insulin treatment was observed for Cp, Lgals3, and Stat3, i.e., expression of those proteins in the insulin-treated group was not significantly different compared to either the control or untreated diabetic group. Fgf2 protein expression remained significantly elevated in the insulin treated group compared to controls. mRNA expression responsiveness was more complex with Lgals3 mRNA levels completely normalized, Cp mRNA levels partially normalized, and Fgf2 and Stat3 levels remaining elevated. C = Control, D = Diabetic, D+I = Diabetic with insulin, * p<0.05, ** p<0.01, one-way ANOVA, Student Newman Keuls post-hoc test.

**Figure 8:**
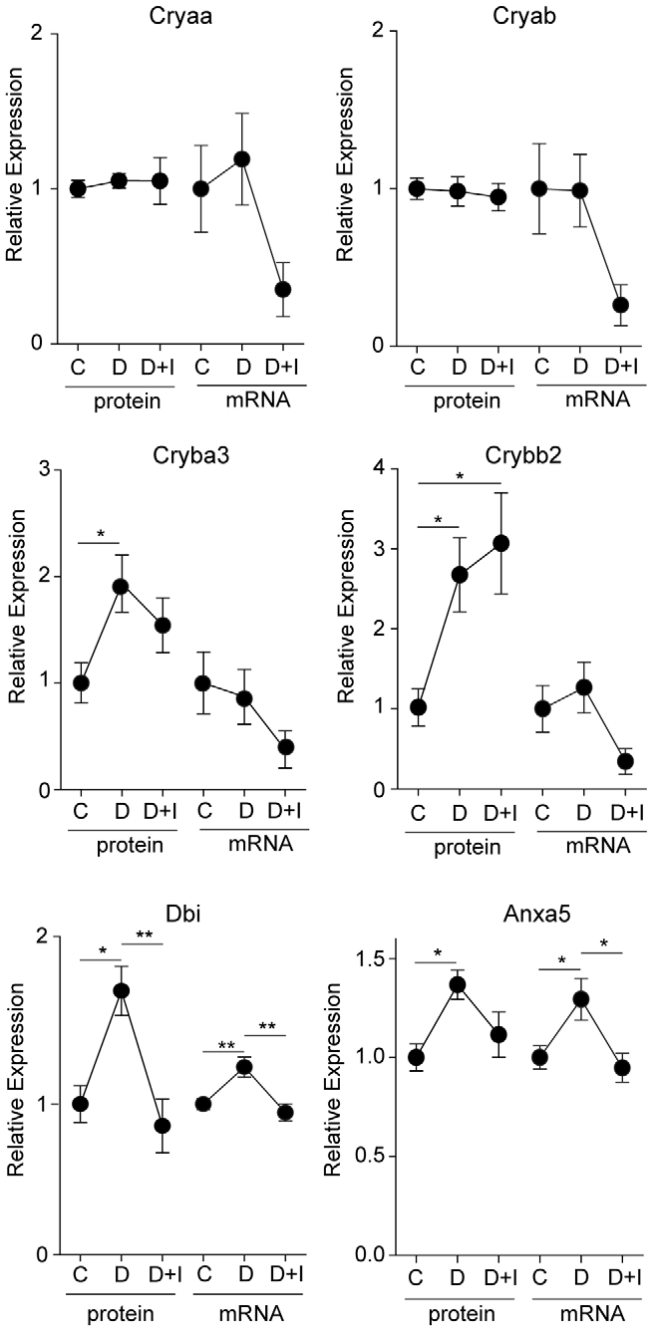
Responsiveness of protein expression to insulin therapy. To examine the effect of insulin treatment on protein and mRNA expression for those targets confirmed in the previous experiments, an independent animal experiment was performed with the addition of an insulin-treated diabetic group that received insulin for 1.5 months after 1.5 months of diabetes without insulin therapy. Increased retinal protein expression of Cryba3, Crybb2, Dbi, and Anxa5 was again observed. Retinal Dbi protein expression was completely normalized by insulin treatment, while Cryba3 protein expression was partially normalized. Crybb2 expression remained elevated following 1.5 months of insulin treatment. The previously observed elevations in Cryaa and Cryab were not recapitulated in this set of animals. C = Control, D = Diabetic, D+I = Diabetic with insulin, * p<0.05, ** p<0.01, one-way ANOVA, Student Newman Keuls post-hoc test.

To test the effect of restoration of normoglycemia by exogenous insulin on retinal protein and mRNA expression, rats received continuous insulin administration by implantation of subcutaneous pellets for the last 1.5 months of the 3 month diabetic period (Experiment 5). Expression of the 10 proteins confirmed in the previous animal experiments was quantified by immunoblotting. A number of different patterns in protein expression were observed, with some changes not normalized by insulin, others partially normalized and some completely returned to control levels. Fgf2 protein and mRNA expression were not normalized by insulin treatment, with levels remaining significantly elevated compared to controls (Figure 7). Insulin treatment partially normalized the protein expression of Cp, Lgals3, and Stat3 with the insulin-treated group no longer significantly elevated from control but also not significantly lower than the diabetic group (Figure 7). The diabetes-induced increases in Dbi and Anxa5 protein and mRNA expression were completely normalized (Figure 8). Crystallin isoform expression demonstrated a variety of patterns. Crybb2 protein expression was not normalized by insulin treatment, while Cryba3 protein expression was partially normalized. Unexpectedly, despite the confirmation of Cryaa and Cryab in previous experiments, no changes in protein expression with diabetes were observed in this experiment.

## Discussion

The results of the combined proteomic, transcriptomic, and confirmation experiments both validate previously reported changes in retinal protein expression with diabetes and identify novel changes. These findings have implications for both our understanding of the pathogenesis of DR and as preclinical drug development biomarkers. Additionally, the combination of discovery approaches used provides comparative insight into commonly used quantitative proteomic techniques.

### Biomarker development

The proteomic profiling and confirmation experiments detailed in this report could be used as the first stages towards developing a panel of protein biomarkers for use in preclinical studies for disease monitoring and evaluation of novel treatment efficacy, similar to previously reported mRNA biomarkers [12]. Further validation is required before these proteins can be used as surrogate endpoints in drug testing programs [12]. Additionally, those proteins not fully normalized by chronic insulin treatment in this study (Fgf2, Cp, Lgals3, Stat3, Cryba3, Crybb2) may be useful in testing novel therapeutics intended to be used in conjunction with insulin therapy to treat those dysregulations caused by diabetes that are not normalized by insulin replenishment.

### Biological functions of confirmed proteins

In agreement with previous reports, diabetes induced retinal expression of multiple crystallin isoforms was observed. Crystallin isoforms Cryaa (α-crystallin A), Crybb2 (β-crystallin B2), Cryab (α-crystallin B) and Cryba3 (β-crystallin A3) were confirmed to be induced after 3 months of diabetes. Interestingly, Cryba3 protein expression was only partially normalized by insulin treatment and Crybb2 protein expression was unaffected.

Crystallin proteins are associated with a variety of functions ranging from lens structure to stress response, but little is known about their potential involvement in diabetic retinopathy. Alpha crystallins, which are similar to small heat shock proteins, have been reported to have neuroprotective effects [31]. Cryab is upregulated in activated astrocytes following ischemic damage [32] and decreases peroxide-induced apoptosis of astrocytes through suppression of caspase-3 signaling [33]. The upregulation of Cryaa and Cryab with diabetes in our work suggests a potential compensatory response in the neural retina that may act to protect neurons and glia from inflammatory insults. Beta crystallins are expressed more highly in the retina than in other non-lens tissues [34], [35], and Cryba3 was recently reported to be restricted to astrocytes in the neural retina [36] Neuroprotection of retinal ganglion cells in a rodent model of ocular hypertension has also been reported to depend on upregulation of Crybb2. Altered expression of crystallin proteins has been previously reported with diabetes, although this is the first study with targeted confirmation analysis of multiple crystallin isoforms. The findings of this work and other literature [23], [37] suggest a protective role of crystallin upregulation in the retina with diabetes. The continued induction of Cryba3 and Crybb2 after restoration of normoglycemia by insulin may be beneficial but also suggests continuing stress to the retina.

Dbi (diazepam binding inhibitor) and Anxa5 (Annexin A5) were identified as targets of interest in our iTRAQ analysis. Dbi is an insulin-regulated transport molecule with numerous functions and protein interactions ranging from regulation of lipid metabolism [38] to binding components of inhibitory neurotransmission machinery [39]. In the retina, Dbi is expressed by pigment epithelium cells and Müller glia [40], and secrete this protein in response to protein kinase C activation [41]. Dbi binds GABA(A) receptors with high affinity [42], [43], and it is thought that suppression of GABAergic signaling is one mechanism through which Dbi modulates retinal neurotransmission, particularly in the inner plexiform layer [40], [41], [44], which further increases following activity-dependent phosphorylation [45], which is a hallmark of diabetes-related retinal pathology [46]. Combined with previous reports of insulin-mediated regulation of Dbi expression [47], [48], the responsiveness of Dbi protein and mRNA to insulin therapy in the present study suggests that this target may be a promising candidate for treatments focused on modulating neurovascular function in the diabetic retina.

Although well-characterized for its utility as a marker of early apoptosis due to its high binding affinity for phosphatidylserine, Anxa5 mediates a variety of cellular functions including adhesion and calcium flux in addition to phosphatidylserine-catalyzed inflammation and apoptosis. This glycoprotein is expressed in vascular cells [49], neurons [50] and in the blood [51] among other locations. In the retina, Anxa5 has been localized to the ganglion cell layer [52], where it may influence neuronal function through binding of the postsynaptic scaffolding plasma membrane protein PSD95 [53].

Our data agree with a previous report of upregulated Fgf2 protein levels after 3 months of diabetes in the Sprague Dawley rat [54]. A novel finding here was that 1.5 months of insulin treatment did not restore Fgf2 protein or mRNA expression to control levels. Previously a sustained induction of Fgf2 mRNA levels in spite of insulin treatment at a one month time point has been reported [55]. Increased retinal Fgf2 protein levels may serve a neuroprotective role [56] and as such, continued induction after insulin therapy may not be deleterious. However, as Fgf2 is induced by a wide range of retinal insults, Fgf2 induction may indicate continued damage to the retina even after restoration of normoglycemia.

Stat3 activation by diabetes and subsequent blood retinal barrier damage can be prevented by simvastatin treatment [57]. The angiotensin converting enzyme inhibitor enalapril has been demonstrated to prevent the diabetes-induced up-regulation of Stat3 protein [58]. Stat3 can exert a large number of effects as a signaling regulator to a number of downstream endpoints. As such, modulation of Stat3 expression likely impacts a variety of pathways and processes in the retina and may prove to be a useful target in drug development efforts for diabetic retinopathy.

Lgals3 (lectin, galactoside-binding, soluble 3; galectin-3) is an abundant protein expressed in the nucleus, cytoplasm, mitochondria, and plasma membrane of multiple cell types including inflammatory macrophages [59], retinal Müller glia [30] and endothelial cells [60]. This protein binds advanced glycation end products (AGEs) with high affinity [61] and, as such, may play an important role in the development of diabetes complications. Lgals3 expression has previously been reported to increase in Muller cells of diabetic rats in association with increased acute phase genes [30]. Lgals3 has also been shown to suppress retinal angiogenesis [62] and promote inner blood-retina barrier dysfunction and breakdown [63]. It is likely that increased Lgals3 expression with diabetes is mediated by increased AGE formation [64], [65] and contributes to inflammatory [66] and vascular components of retinal complications.

Likewise, ceruloplasmin (Cp), an iron binding and metabolizing α2 globulin, was upregulated with diabetes in this work. This protein is a component of the acute phase response, and is induced by inflammation [67], [68] as well as interleukin-1 stimulation [69], although Cp upregulation appears to constitute an anti-inflammatory cytoprotective response [67]. Cp protein is increased in sera from hyperglycemic patients [70] and after optic nerve crush [71]. Cp transcript is also increased in retinal Müller cells of diabetic rats [30] which may secrete this protein and ultimately impact retinal microvasculature. It has been proposed that upregulation of Cp protects against oxidative damage by decreasing ferritin levels and reducing AGE-mediated damage [72]. Given the potential protective roles of these proteins, further study is required before a definitive determination of the benefits of normalizing their expression with insulin can be made.

Previously, in focused studies examining specific proteins changes in expression of para-inflammatory and microvascular-related proteins (e.g., Vegf, Icam1, TNFα, and IL-1β) have been reported in rodent models of DR. Some of these proteins, such as Icam1, were not observed with any of the three proteomic methods used in this study. For other proteins, no changes in expression were observed (e.g., Vegf, TNFα, and IL-1β). Increased Vegf protein expression has been reported in the Sprague Dawley STZ model at after 1 and 6 months of diabetes [73], [74] but not after 3 months [54]. Similarly varying results have been reported for the induction of both Il-1β and TNFα depending on the duration of diabetes [54], [75]–[77]. Clearly, there are important temporal characteristics to the expression of these proteins with diabetes. The DR field would greatly benefit from a thorough characterization of the expression and localization of these proteins across different durations and in multiple rodent models of diabetes, expanding on work previously reported by Kirwin and colleagues [54].

### Comparison of proteomic technologies

The overarching finding from the comparison of the methods used in this study is that the different techniques implemented are complementary rather than redundant. Comparison of the biophysical properties of proteins identified by the different methods demonstrated no preferential discrimination of protein molecular weights between iTRAQ and DIGE. However, the distribution of identified iTRAQ proteins was skewed toward hydrophilic proteins while those identified by the DIGE analysis were more hydrophobic. Proteins detected by the Luminex analysis were evenly distributed across molecular weights and hydropathicity. Since Luminex assays are predesigned to select specific proteins, the technical capabilities of this assay are highly dependent on the specific antibodies used. The inclusion of transcriptomic analysis provided novel targets not observed in any of the proteomic analyses. Interestingly, all targets chosen from the transcriptomic analysis were confirmed at both the mRNA and protein levels. In total, the comparison of mRNA and protein expression levels demonstrates that while mRNA changes are not determinative of protein expression changes in many cases they are highly correlated.

This study identified a number of retinal protein expression changes with diabetes. Of particular interest for future studies are those proteins that are not normalized (Fgf2 and Crybb2) or only partially normalized (Cp, Cryba3, Lgals3, Stat3) by insulin treatment, as these persistent changes reflect the limitations of insulin to restore the retina to a nondiabetic state. The lack of complete normalization by insulin treatment offers the potential that these proteins may play a role in the ‘metabolic memory’ [78] observed clinically in retinopathy pathogenesis [79]. Future studies will address when these protein changes first occur as well as the cellular origin of these proteins and the mechanisms underlying their continued dysregulation despite insulin replacement.

## Materials and Methods

### Ethics Statement

All animal experimental procedures were approved by and carried out in accordance with the Penn State College of Medicine Institutional Animal Care and Use Committee (Approval ID Number: 2006–093). Animal surgery and sacrifice was performed under sodium pentobarbital anesthesia, and all efforts were made to minimize suffering.

### Animal Methods

All rats were maintained by the Penn State JDRF Animal Models Core in accordance with the Penn State College of Medicine Institutional Animal Care and Use Committee guidelines under specific pathogen-free conditions and monitored by quarterly sentinel testing. Diabetes was induced in male Sprague-Dawley rats (Charles River Laboratories, Wilmington, MA) (100–125 g at arrival) by intraperitoneal streptozotocin as previously described [14]. Control rats were injected with an equal dose of vehicle. Only STZ-injected rats with blood glucose levels >250 mg/dL throughout the experiment were included in this study (Table 1). No exogenous insulin was delivered in animal experiments 1-4. For animals in experiment 5, the insulin treatment group received one 26 mg subcutaneous pellet (LinShin Canada, Scarborough, Canada) delivered via trocar 6 weeks post-STZ injection. An additional 26 mg implant was introduced when body weight exceeded 300 g or when midday non-fasting blood glucose exceeded 250 mg/dL. Blood glucose monitoring, animal sacrifice, and retina excision were conducted as previously described [12]. Glycosylated hemoglobin was measured from drops of blood acquired by nicking the tail, using a Lifescan glucose meter and Siemens DCA Analyzer, respectively.

### DIGE

Quantitative two-dimensional in-gel electrophoresis (DIGE) was performed as detailed previously [80], [81]. All DIGE methods and results are provided in MIAPE-GE compliant form [82]. Retinas [control, n = 8; diabetic, n = 8; (Experiment 1, Table 1)] were homogenized in lysis buffer, and soluble proteins were purified, quantitated, and labeled with Cy3 and Cy5 dyes as previously described [81]. A normalization pool containing equal protein from each sample, for inclusion on each 2D gel, was labeled with Cy2. 250 µg of unlabeled protein from a representative pool containing equal protein from all samples was used for a preparative/picking gel for mass spectrometry (MS) identification of protein species. First and second dimension separation was performed as previously described [80], [81]. All gels were imaged with a Typhoon 9410 scanner (GE Healthcare) and analyzed using semi-automated DeCyder 6.5 software (GE Healthcare).

For each protein spot, the ratio of background-subtracted sample-specific signal (Cy3 or Cy5 density) to normalization pool signal (Cy2) was calculated. Only protein spots detected on ≥6/8 analytical gels were included in quantitation analysis. One-way ANOVA (p<0.05) and a 1.1-fold change cutoff were used to determine protein spots significantly regulated with diabetes.

### MALDI-ToF/ToF Mass Spectrometry

MALDI-ToF/ToF mass spectrometry was performed as described previously [81], [83]. Spots of interest were excised from the picking gel using a robotic Ettan Spot Picker (GE Healthcare), and gel plugs were subjected to in-gel trypsin digestion and ZipTip (Millipore, Bedford, MA) desalting. Protein identifications were performed by peptide mass fingerprinting (MS and MS/MS) using standard laboratory methods and a Applied Biosystems 4800 Proteomics Analyzer in reflector positive mode [80], [81]. An exclusion list containing autolytic tryptic peptides, human keratin, matrix, and precursor peptides identified in a “blank” gel plug was included as an additional filter. Data interpretation was performed using GPS explorer (v3.6) and database searching was carried out using MASCOT (v2.0.00). Database searches were carried out against the *Rattus* taxonomy of the NCBI database downloaded on Feb 16 2008 (107,758 entries searched). In the final combined MS and MS/MS database searches, identifications required a MASCOT confidence interval ≥95%.

### iTRAQ analysis

8-Plex iTRAQ (Applied Biosystems) sample preparation and labeling were performed according to manufacturer's instructions as previously described [23] with samples from Experiment 1 (Table 1). Following purification by acetone precipitation, 100 µg of retinal protein from each of four control and four diabetic rats was reconstituted in dissolution buffer (0.5 M TEAB, pH 8.5), denatured with 2%SDS, and alkylated/reduced with 5 mM TCEP/20 mM MMTS. Trypsin digestion was conducted overnight at 37°C by incubation with sequencing-grade trypsin prior to labeling with 8-plex iTRAQ reagents. The eight labeled samples were combined and prepared in 10 mM KH_2_PO_4_ for LC-SCX separation. SCX separations were performed on a passivated Waters 600E HPLC system, using a PolySULFOETHYL Aspartamide column (PolyLC, Columbia, MD) prior to reverse phase C18 nanoflow-LC separation performed using a Tempo LC MALDI Spotting System (ABI-MDS/Sciex) and a Chromolith CapRod column (Merck).

MALDI target plates were analyzed in a data-dependent manner on an ABI 4800 MALDI TOF-TOF. Both MS and MS/MS default calibrations were performed using the Applied Biosystems Calibration Mixture 1. MS Spectra were acquired from each sample spot and selected peaks were analyzed by MS/MS with collision-induced decay.

Protein identification and quantitation were performed using the Paragon algorithm as implemented in Protein Pilot 2.0 software (ABI/MDS-Sciex). Database searches were carried out against the *Rattus* subset of the NCBI database downloaded on July 22 2008 (109,096 entries searched) with a concatenated with a reversed “decoy” version. Confident protein identifications required a ProteinPilot Unused Score of at least 1.3 (95% confidence interval) and a Local False Discovery Rate estimation with a decoy database of no higher than 5% using PSPEP software [84].

### Luminex

Retina protein profiling was performed at Rules-Based Medicine (Austin, Texas) using standard Luminex technology [27]. Retinal samples (in triplicate) from Experiment 1 (Table 1) (n = 8/group) were subjected to RodentMap antigen analysis for 59 different circulating proteins. Samples were mixed with capture microspheres, incubated with multiplexed biotinylated reporter antibodies, and developed with streptavadin-phycoerythrin. Analysis was performed in a Luminex 100 instrument, with analytes quantitated using 4 and 5 parameter, weighted and non-weighted curve fitting algorithms included in the data analysis package.

### RNA Isolation

Retina homogenization and RNA isolation was performed by standard Tri Reagent/BCP disruption and phase separation followed by over-night isopropanol precipitation at -20°C, as previously described [14]. After purification using Qiagen RNeasy Mini kit (Qiagen), RNA was assessed for quantity and quality using a NanoDrop ND1000 (Thermo Scientific, Wilmington, DE) and RNA 6000 Nano LabChip with an Agilent 2100 Expert Bioanalyzer (Agilent, Palo Alto, CA), respectively.

### Microarray analysis

Microarray analysis was performed with Illumina RatRef12 microarrays (Illumina, San Diego, CA) according to standard procedures [15] using five control and five diabetic rats from Experiment 2 (Table 1). 500 ng RNA was reverse transcribed to first strand cDNA by incubation with T7 Oligo(dT) primer, 10× First Strand buffer, dNTPs, Rnase inhibitor, and ArrayScript. Second strand cDNA was synthesized with 10× second strand buffer, dNTPs, DNA polymerase and RNase H. Purified cDNA was eluted in nuclease-free water and *in vitro* transcribed to synthesize cRNA by incubation with T7 10× Reaction buffer, T7 Enzyme mix and Biotin-NTP mix. cRNA was purified according to manufacturer's instructions and quantitated using a NanoDrop ND1000 spectrometer. 750 ng of purified cRNA was prepared for hybridization and incubated with RatRef-12 BeadChips prior to washing and staining with streptavadin-Cy3 stained. BeadChips were then dried and subsequently scanned using a BeadStation BeadArray Reader.

After initial quality control of arrays, one array from the diabetic group was removed. GenomeStudio (Illumina) flat files were imported into GeneSpring GX11 (Agilent) software for data analysis. Using the detection p-values initially generated in GenomeStudio, probes were required to have detectable signal (marginal or present calls) in all arrays for at least one of the two groups in the experiment to be included in the subsequent statistical analyses. This filtering eliminates from further analysis transcripts not reliably detected, while retaining genes potentially expressed in only one group (i.e., expressed only in control or only in diabetic). The full complement of microarray data is available in the Gene Expression Omnibus [85], accession # GSE20886.

To determine differential expression, a combination of statistical p value (two-tailed t-test, p<0.05) and fold-change (≥1.2 fold) cutoffs were used in accordance with standards in data analysis [86] and our previously published methods [14], [15]. Differential mRNA expression data were used to generate a heatmap representation of retinal transcriptomic profiles. The heatmap was organized by up- and down-regulated expression segregated by horizontal brackets. Individual animals were clustered by Euclidean distance using K-means with complete linkage hierarchy (vertical brackets).

### Quantitative RT-PCR

Quantitative PCR confirmations were performed as described previously [12], [14] using an 7900HT Sequence Detection System (Applied Biosystems, Foster City, CA), 384-well optical plates, and Assay-On-Demand (Applied Biosystems) gene specific primers and probes (Experiments 3 and 5, n = 8–9/group, Table 1). SDS 2.2.2 software and the 2^−ΔΔCt^ analysis method were used to quantitate relative amounts of product using β-actin as an endogenous control [12]. β-actin levels were determined to be unchanged in an absolute quantitation experiment (data not shown). For a full listing of primer/probe sets see Table S5.

### Immunoblot analysis

Soluble retinal protein was isolated from independent sets of animals (Experiments 3–5, n = 8–10/group, Table 1) by homogenization in 250 µL detergent-based protein lysis buffer (2.5 mM HEPES, 1 mM EDTA, 100 mM NaCl, 1 mM dithiothreitol, 1% Tween 20, 1 mM Na_3_VO_4_, 2 protease inhibitor tablets/15 mL). Insoluble material was removed by centrifugation at 10,000×g for 10 minutes at 4°C. Protein concentrations were determined by Pierce BCA reagent. Samples were separated on Criterion 4–20% gradient gels (Bio-Rad) and transferred to either PVDF or LF-PVDF membranes (GE Healthcare) depending on the manner of subsequent visualization (chemiluminescence or fluorescence). Membranes were incubated with primary and secondary antibodies as described in Table S6. Images were analyzed with ImageQuant TL (GE Healthcare).

### Statistical Analysis

For all immunoblot and qPCR experiments, the data for individual samples were normalized to the respective actin signal. Statistical analyses were conducted using SigmaStat 3.5 software, with two-tailed t-tests for two group comparisons (control vs. diabetic) and one-way ANOVA with pair-wise Student Newman Keuls post-hoc tests for multiple group comparisons.

### Bioinformatic Analysis

Coverage and differential expression between proteomic and transcriptomic approaches were evaluated. Protein accession numbers and gene IDs were converted to gene symbols using Ingenuity Pathway Analysis (IPA) software to eliminate potential redundancy and incomplete mapping of identifiers. Proteins with multiple identifications in a method (e.g., multiple spots with the same protein ID in the DIGE experiment) were compressed to one unique gene symbol. Overlap between Luminex, DIGE, and iTRAQ coverage (i.e., unique detected/identified proteins) was visualized by Venn diagram. Total proteomic coverage (i.e., the union of the three proteomic approaches) was then compared to transcriptomic coverage. Similarly, Venn diagrams were created to illustrate commonalities and differences in differentially regulated species detected by proteomic and transcriptomic methods.

The biophysical properties of detected/identified proteins for each proteomic method (Luminex, DIGE, iTRAQ) were determined. NCBI gi| accession numbers were translated to UniProt/Swiss-Prot accession numbers and imported into the Swiss Institute of Bioinformatics Expert Protein Analysis System (ExPASy) ProtParam tool (http://www.expasy.org). Molecular weights and grand average of hydropathicity (GRAVY) scores were retrieved for each unique protein, and binned according to size (0 kDa to >150 kDa) and score (<−1.0 to >0.4) for comparison of frequency distributions.

## Supporting Information

Figure S1 **Whole-genome gene expression analysis.** Retinal RNA from control (n = 5) and diabetic (n = 4) rats collected after 3 months of hyperglycemia was analyzed by Illumina Rat Ref12 microarray. 11,874 of 22,517 probes on the microarray had detectable signals. The 1,274 probes with significantly different expression between control and diabetic groups (two-tailed t-test, p<0.05, 1.2 fold cut-off), including 583 down-regulated and 691 up-regulated genes, are shown.
(TIF)

Figure S2 **Comparison of biophysical properties of identified proteins.** The biochemical characteristics of proteins identified by the three complementary proteomic methods are illustrated by frequency plots. (**A**) Molecular weight distributions of unique species identified in iTRAQ, DIGE and Luminex approaches are depicted as the percent of the total protein coverage for each method. Proteins identified in iTRAQ and DIGE were similarly distributed across a broad molecular weight range, while the directed Luminex approach provided better coverage of smaller (10–30 kDa) species. (**B**) The relative hydrophobicity/hydrophilicity of proteins identified by the three approaches were compared using grand average of hydropathicity (GRAVY) scores for each protein. All three methods provided broad coverage ranging from hydrophilic (negative score) to hydrophobic (positive score) proteins. A greater percentage of the proteins observed in the iTRAQ analysis were hydrophilic in nature while DIGE or Luminex approaches had a greater percentage of hydrophobic proteins, further demonstrating the complementarity of the three approaches.
(TIF)

Table S1 **Primary data from Luminex analysis**
(XLS)

Table S2 **Primary data from DIGE analysis**
(XLS)

Table S3 **Primary data from iTRAQ analysis**
(XLS)

Table S4 **Differentially expressed genes from transcriptomic analysis**
(XLS)

Table S5 **Primer/probe sets used in qPCR confirmations**
(DOC)

Table S6 **Antibodies used in immunoblot confirmations**
(DOC)
