# Supplementary figures and images for: Multi-Modal Proteomic Analysis of Retinal Protein Expression Alterations in a Rat Model of Diabetic Retinopathy
Source: PLoS One. 2011 Jan 13;6(1):e16271. doi: 10.1371/journal.pone.0016271 (PMC3020973; doi:10.1371/journal.pone.0016271)

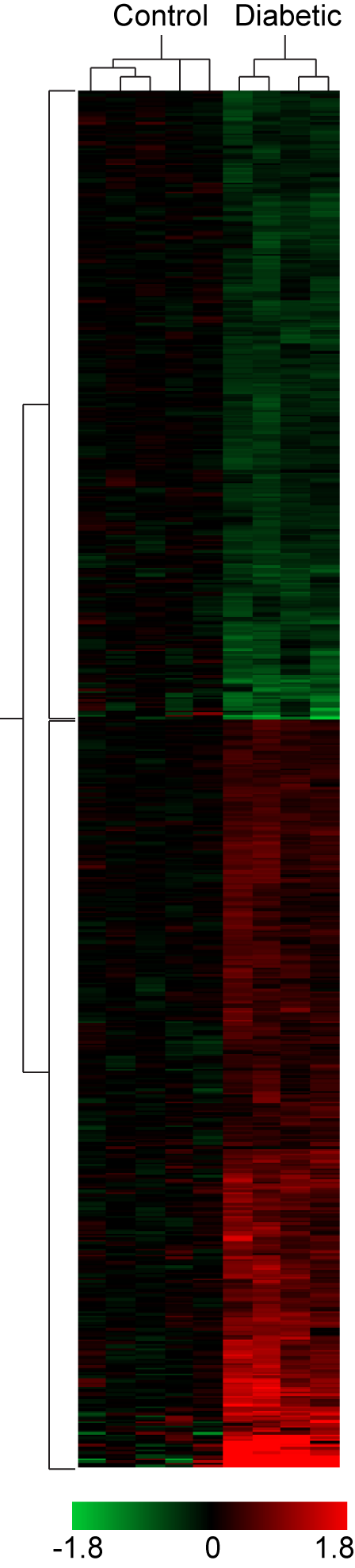

Supplement: Figure S1 — Whole-genome gene expression analysis. Retinal RNA from control (n = 5) and diabetic (n = 4) rats collected after 3 months of hyperglycemia was analyzed by Illumina Rat Ref12 microarray. 11,874 of 22,517 probes on the microarray had detectable signals. The 1,274 probes with significantly different expression between control and diabetic groups (two-tailed t-test, p<0.05, 1.2 fold cut-off), including 583 down-regulated and 691 up-regulated genes, are shown. (TIF) [file pone.0016271.s001.tif]

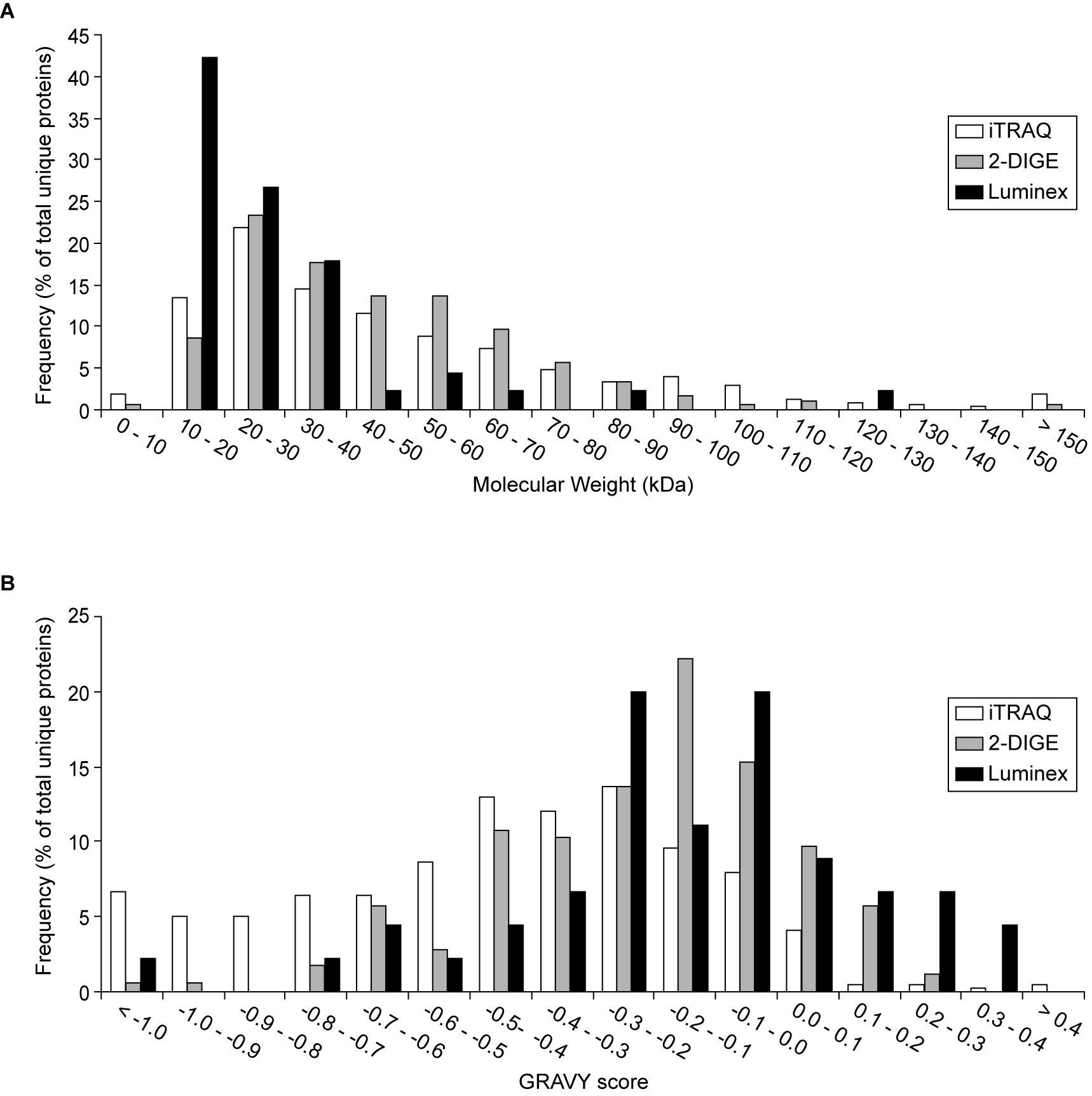

Supplement: Figure S2 — Comparison of biophysical properties of identified proteins. The biochemical characteristics of proteins identified by the three complementary proteomic methods are illustrated by frequency plots. (A) Molecular weight distributions of unique species identified in iTRAQ, DIGE and Luminex approaches are depicted as the percent of the total protein coverage for each method. Proteins identified in iTRAQ and DIGE were similarly distributed across a broad molecular weight range, while the directed Luminex approach provided better coverage of smaller (10–30 kDa) species. (B) The relative hydrophobicity/hydrophilicity of proteins identified by the three approaches were compared using grand average of hydropathicity (GRAVY) scores for each protein. All three methods provided broad coverage ranging from hydrophilic (negative score) to hydrophobic (positive score) proteins. A greater percentage of the proteins observed in the iTRAQ analysis were hydrophilic in nature while DIGE or Luminex approaches had a greater percentage of hydrophobic proteins, further demonstrating the complementarity of the three approaches. (TIF) [file pone.0016271.s002.tif]
