# Supplementary material for: Multi-Modal Proteomic Analysis of Retinal Protein Expression Alterations in a Rat Model of Diabetic Retinopathy
Source: PLoS One. 2011 Jan 13;6(1):e16271. doi: 10.1371/journal.pone.0016271 (PMC3020973; doi:10.1371/journal.pone.0016271)
Supplement: Table S5 — Primer/probe sets used in qPCR confirmations (DOC) [file pone.0016271.s007.doc]

Table S5. Primer/Probe Sets

| **TaqMan Target Assay** | **Applied Biosystems Assay ID** |
| --- | --- |
| Actin | Rn00667869_m1 |
| Ceruloplasmin | Rn00561049_m1 |
| Crystallin-Aa | Rn00561064_m1 |
| Crystallin-Ab | Rn00564026_m1 |
| Crystallin-Ba3/A1 | Rn01496018_m1 |
| Crystallin-Bb2 | Rn00564035_m1 |
| FGF-basic | Rn00570809_m1 |
| Galectin-3 | Rn00582910_m1 |
| Stat3 | Rn00562562_m1 |
| Acbp | Rn00821402_g1 |
| Annexin V | Rn00565571_m1 |
