# Supplementary material for: Multi-Modal Proteomic Analysis of Retinal Protein Expression Alterations in a Rat Model of Diabetic Retinopathy
Source: PLoS One. 2011 Jan 13;6(1):e16271. doi: 10.1371/journal.pone.0016271 (PMC3020973; doi:10.1371/journal.pone.0016271)
Supplement: Table S6 — Antibodies used in immunoblot confirmations (DOC) [file pone.0016271.s008.doc]

| **Protein** | **Vendor** | **Cat#** |
| --- | --- | --- |
| Actin | Chemicon | MAB1501 |
| Ceruloplasmin | Novus | NB600-1348 |
| Crystallin - Aa | SCBT | sc-28306 |
| Crystallin - Ab | Novus | NB120-13496 |
| Crystallin - Ba3/A1 | SCBT | sc-22398 |
| Crystallin Bb2 | SCBT | sc-22409 |
| FGF- basic | BD Biosciences | 610871 |
| galectin 3 (lgals3) | SCBT | sc-19283 |
| STAT3 | BD Biosciences | 610189 |
| Acbp / DBI | SCBT | sc-23474 |
| Annexin V | SCBT | sc-8300 |

Table S6. Antibodies
